# Learning from neurodivergent students about their mental health and wellbeing during transition to secondary school: A systematic review

**DOI:** 10.1002/jcv2.70175

**Published:** 2026-10-20

**Authors:** Olympia Palikara, Rebecca Lucas, Danielle Baptiste, Cat Jones, Laura Kelly, Georgia Pavlopoulou, Vassilis Sideropoulos

**Affiliations:** ^1^ Department of Education Studies University of Warwick Coventry UK; ^2^ Department of Psychology Royal Holloway University of London Egham UK; ^3^ Group for Research in Relationships and Neurodiversity (GRRAND), Division of Psychology & Lang Sciences, Department of Clinical, Educational & Health Psychology Faculty of Brain Sciences University College London London UK; ^4^ Anna Freud Centre London UK; ^5^ Department of Psychology and Human Development IOE UCL's Institute of Education University College London London UK

**Keywords:** mental health, neurodiversity, primary to secondary education, systematic review, transition, wellbeing

## Abstract

The impact of transition from primary to secondary education for neurodivergent children is an area where research, though emerging, has not yet produced consistent or conclusive findings. By adopting a neurodiversity‐informed framework, we conducted a systematic review of empirical studies examining the child‐reported impact of this transition on psychosocial and mental health outcomes amongst neurodivergent children such as autistic, children with developmental language disorder, and children with Down syndrome (DS). Six databases (PubMed, PsycInfo, Scopus, Web of Science, ASSIA, and Google Scholar) were searched from 1995 to July 2026. The review followed the preferred reporting items for systematic reviews and meta‐analyses (PRISMA) guidelines for transparent reporting. Of the 488 records identified, only 10 studies met the inclusion criteria: longitudinal empirical studies with comparison groups, including minimum two timepoints before and after the transition from primary to secondary school, with child‐reported psychosocial, mental health, or wellbeing data. Study quality was assessed using the quality appraisal checklist. Given substantial heterogeneity across studies, a narrative synthesis following synthesis without meta‐analysis (SWiM) guidelines was conducted. Key gaps in the existing literature include the near‐total absence of co‐produced or participatory research designs, underrepresentation of specific neurodivergent groups (e.g., children with Williams syndrome and DS), and failure to examine intersectional factors such as ethnicity and socioeconomic status as moderators of transition‐related outcomes. Findings on the impact of transition on mental health were mixed and inconclusive, while psychosocial outcomes such as school concerns, belonging, and connectedness, emerged as more sensitive and consistent indicators of transition‐related vulnerability. Methodological priorities for future research include longitudinal co‐produced designs, intersectional analyses, and inclusive outcome frameworks developed with neurodivergent children themselves. The theoretical and educational implications of this review for the development of evidence‐based, neurodiversity‐affirming intervention studies targeting the transition period are discussed.

## Key Points

**What's Known?**

- Transition‐related mental health challenges are influenced more by environmental fit and support than intrinsic vulnerabilities.

**What's New?**

- Transition‐related psychosocial outcomes (e.g., school concerns, belonging, connectedness) emerged as more sensitive and consistent indicators of vulnerability than diagnostic mental health measures.
- Experiences vary across neurodivergent groups; underrepresented populations require focused study.
- Methodological factors, including the design of the study, the timing of the follow‐up time‐point, and whether child or parent/teacher perspectives were reported largely shaped observed outcomes.

**What's Relevant?**

- There is a critical need for international, longitudinal, participatory, neurodiversity‐affirming research to inform inclusive, context‐responsive transition support and intervention design.

## INTRODUCTION

The transition from primary to secondary school represents a key developmental and educational milestone, offering both new opportunities and challenges. It typically occurs around the age of 11 and coincides with the onset of puberty, a period associated with substantial cognitive, socio‐emotional, and personality development (Mandy et al., 2016; Sideropoulos et al., 2024; Thompson et al., 2003). This convergence of educational and developmental changes also aligns with the emergence of anxiety and depression during early adolescence (Thapar et al., 2022). These overlapping transitions may place both neurotypical and neurodivergent students at heightened risk of developing mental health conditions, a trend exacerbated during the COVID‐19 pandemic (Castle et al., 2024). Yet dominant responses to these challenges have often framed neurodivergent children's experiences as individual deficits, rather than as products of unsupportive environments.

Our current review, situated within the neurodiversity paradigm, challenges deficit‐based and medicalised accounts that have historically contributed to stigma and exclusion (Botha et al., 2023; Cheng et al., 2025; Pavlopoulou et al., 2025). Rather than conceptualising neurodivergences such as autism, dyslexia, attention deficit hyperactivity disorder (ADHD), developmental language disorder (DLD), language disorder (LD) and Down syndrome (DS) as deficits to be remediated, the neurodiversity paradigm recognises neurological differences as natural variations in human cognition. From this perspective, psychosocial (e.g., sense of belonging, school connectedness, friendships) and mental health (e.g., anxiety, depression, stress) outcomes are understood not solely as characteristics of the individual but also as qualities shaped by the fit between individuals and their social and educational environments, including support and adjustments available within schools and within their immediate environment. The aforementioned neurodivergences are amongst the most prevalent within school settings (Cai et al., 2025).

Within educational contexts, the transition to secondary school comes with substantial changes in school structure, peer networks, expectations, and levels of support. Research suggests that this period can present challenges for many neurodivergent students, particularly amid substantial changes in the school environment, expectations and support systems (Makin et al., 2017; Mandy et al., 2016). Some studies have highlighted differences in school experiences depending on type of provision and environment. For example, autistic students in mainstream schools in the UK recently reported significantly higher emotional burden at school compared to their neurotypical peers (Pavlopoulou et al., 2025), which was driven by both frequent exposure to upsetting events (e.g., imposed control; sense of injustice, alienation) within school contexts and stronger emotional responses to those experiences. Studies from Australia, the United States, and Ireland report that increased academic demands, larger and less predictable school settings, and reduced continuity with familiar teachers can intensify anxiety and adjustment challenges for autistic students and those with ADHD or learning disabilities (Jindal‐Snape et al., 2020; Knesting et al., 2008).

Understanding why neurodivergent students face amplified challenges during this transition requires attention to the school environment itself, rather than solely to individual characteristics. Changes in school environments, expectations and social contexts during transition may influence children's experiences of belonging, support and wellbeing, particularly where there is a mismatch between students' needs and the support available in the new school environment (Keen et al., 2016; Norbury, Gooch, Baird, et al., 2016, Norbury, Gooch, Wray, et al., 2016). Eccles et al. (1993) proposed that declining motivation and wellbeing during school transition reflects a poor fit between the developing needs of students and the environments they encounter, rather than deficits within the students themselves. That misfit is often compounded by the social demands of larger, less structured environments—including navigating peer hierarchies, unstructured social time, and unwritten rules—which can be particularly challenging for neurodivergent students (Horgan et al., 2023; Humphrey & Symes, 2010; McKinlay et al., 2025). Yet despite this, teacher awareness and preparedness to support neurodivergent students remains inconsistent, with many teachers reporting limited training in identifying and responding to neurodivergent needs within mainstream setting (Horgan et al., 2023; McKinlay et al., 2025). This suggests that barriers neurodivergent students face at transition to secondary school are as much a product of how schools are structured and staffed as they are of individual neurodevelopmental profiles.

Evidence from Ireland further indicates decline in students' sense of social belonging and engagement during this transition, particularly when support structures are inconsistently implemented across school levels (Foley et al., 2016). Research from Australia similarly highlights that the loss of trusted relationships can exacerbate stress, highlighting the need for coordinated transition planning (Wilmot et al., 2023). For example, Evangelou et al. (2008) found that 58% of neurodivergent children had difficulties settling in their UK secondary school. These findings have been replicated by Sideropoulos et al. (2024), for autistic children and those with DS and Williams Syndrome. Research from Australia on dyslexia similarly highlights how psychosocial outcomes, such as school connectedness, are shaped by the fit between the student and their educational environment, including access to support in the final year of primary school (Wilmot et al., 2023, 2024). Collectively, international findings emphasise that neurodivergent students face distinct and often amplified barriers during this educational shift.

There is clear evidence that children's attainment and motivation often dip following transition to secondary school, a pattern often attributed to changes in school structure, expectations, and educational support during this period (Symonds & Galton, 2014; West et al., 2010). However, research into the effect of this transition on neurodivergent children's mental health and wellbeing has provided mixed results, with some studies suggesting generally negative experiences (e.g., increased anxiety, distress) amongst different context and neurodivergences (Makin et al., 2017; Tobin et al., 2012; Sideropoulos et al., 2024; Wilmot et al., 2023, 2024) and others reporting some positive changes (e.g., greater routine, more varied lessons) associated with the move to secondary schools (Neal & Frederickson, 2016; Stack et al., 2020). To advance our understanding of how neurodivergent students are affected by the transition to secondary school, we need to also consider the intersections of age, gender, ethnicity, socio‐economic status (SES) and co‐occurring conditions. Mental health and psychosocial outcomes are shaped by those dimensions and considering them improves our understanding, helps us highlight who's at greater risk and allows us to move beyond a diagnostic model in explaining mental health (Cheng et al., 2025; Villatoro et al., 2018). Central to this are also the self‐reports on mental health and wellbeing of neurodivergent children and young people to inform both research and practice (Fletcher‐Watson et al., 2021).

Despite growing interest in the topic, several limitations within the existing literature make it difficult to draw clear conclusions. Many studies rely on very small sample sizes which makes it difficult to generalise the findings (Mandy et al., 2016; Sideropoulos et al., 2024). This challenge is further compounded by the heterogeneity of neurodivergent profiles and the wide variation in school environments. Furthermore, while qualitative data can be rich and offer valuable insights into lived experiences, there are not many studies that looked at the experiences, needs and identification of areas for change to improve psychosocial and mental health outcomes of neurodivergent students during transition to secondary school. All of the above makes it hard to identify what works best and for whom. Furthermore, many of the studies in the area are cross‐sectional and retrospective (Hughes et al., 2013; Maras & Aveling, 2006), making it difficult to reliably examine changes over time. Finally, there is limited data collected directly from neurodivergent children themselves during transition from primary to secondary school, with many studies relying on data collected from parents and/or teachers, despite increasing recognition of the importance of child‐led research and practices (Gondek et al., 2016; Hindley & Whitaker, 2017; Stack et al., 2021). Understanding the self‐reported views of those neurodivergent children is key in order to develop mechanisms and support systems that target the children's needs. It is evident that the literature has overlooked the broader contextual factors, with the majority of research focusing on diagnostic labels and other person‐level factors (e.g., intelligence, cognitive skills). For example, recent work in the ADHD field has similarly highlighted a predominance of person‐level predictors in explaining academic achievement in primary and secondary school, with limited research examining contextual factors such as provision type, study environments and school support (Zhang et al., 2026). This pattern is not unique to ADHD and is evident across research on neurodiversity more broadly. Consequently, factors such as school climate, sense of belonging and connectedness remain comparatively unexplored in informing our knowledge about transitions to secondary schools.

By systematically reviewing longitudinal studies that collected data directly from neurodivergent students alongside appropriate comparison groups, this review aims to generate a robust evidence base on how transition from primary to secondary school affects neurodivergent students' mental health and wellbeing, providing theoretical and empirical foundations necessary to inform future intervention design, inclusive policy, and equitable research and practice. To do so, we address the following three questions: (a) what is the impact of the transition from primary to secondary school on mental health outcomes in neurodivergent students? (b) what is the impact of the transition to secondary school on the psychosocial outcomes relating to transition in neurodivergent students? and (c) are patterns evident within or between specific groups of neurodivergent students?

## METHOD

The present systematic review was conducted in accordance with the preferred reporting items for systematic reviews and meta‐analyses (PRISMA) guidance, and a protocol was registered in the open science framework (OSF) (see Sideropoulos et al., 2025). Reflecting our positionality and commitment to the neurodiversity paradigm as researchers and practitioners, a neurodiversity‐affirming approach was adopted in the interpretation of the results which allows us to discuss psychosocial wellbeing and mental health outcomes in terms of environmental fit, inclusion, agency, and support, rather than normative developmental benchmarks. Within our inclusion criteria, neurodivergent children were defined as those with a formal clinical diagnosis of a neurodevelopmental condition (e.g., autism, ADHD, DLD, LD, DS, dyslexia, or learning disability), or those identified through standardised assessment as meeting criteria for a neurodevelopmental profile. Studies relying solely on self‐identification without corroborating diagnostic or assessment data were not included. This decision reflects the available literature rather than a hierarchy of epistemic validity—we recognise that self‐identification is a legitimate and meaningful form of knowledge, and we acknowledge this as a limitation of the existing evidence base.

Throughout the synthesis, emphasis was placed on identifying participatory methods in research and contextual factors within the school transition process that either supported or constrained children's wellbeing, avoiding pathologizing language and deficit‐based interpretations of children's experiences. This interpretive stance is consistent with growing recognition that psychological and mental health research has too often bracketed out context, focusing on within‐person variables at the expense of the environments, relationships, and structural conditions that shape outcomes (Vella et al., 2023).

### Inclusion criteria

Predetermined inclusion criteria were (1) participants included neurodivergent children aged between 9 and 13 years old (capturing the transition to secondary school internationally), (2) sample included a comparison group (i.e., neurotypical children or other neurodivergent (ND) children), (3) data on psychosocial outcomes, mental health, or wellbeing^1^ was collected before and after the transition from primary to secondary school (e.g., longitudinal studies, or studies with minimum of two timepoints, pre‐ and post‐secondary school transition), (4) the experience of transition from primary to secondary school was measured for example, satisfaction with new provision, (5) empirical data was collected directly from ND individuals, (6) means, standard deviations and sample sizes were reported, (7) published as a peer reviewed journal article and (8) written in English.

Exclusion criteria were as followed: (1) did not collect data on psychosocial outcomes, mental health, or wellbeing both before and after the transition from primary to secondary school. (2) Failed to measure the experience of the transition (e.g., no data on satisfaction or adjustment to new school provision). (3) Relied solely on proxy reports (e.g., from parents or teachers) without direct data from neurodivergent individuals. (4) Did not report necessary statistical information (means, standard deviations, or sample sizes) required for analysis. (5) Were unpublished reports, theses, conference abstracts, or grey literature. (6) Were not written in English.

### Search strategy

Six online databases (PubMed, PsycInfo, Scopus, Web of science, ASSIA, and Google scholar) were searched for relevant articles using a combination of different query strings (see the protocol for search strategy: Sideropoulos et al., 2025). The searches covered studies published from 1995 to 2026. The last search was conducted July 2026. The decision to include studies published from 1995 onwards reflects the period during which systematic empirical research on primary‐to‐secondary transition in neurodivergent children began to emerge in the peer‐reviewed literature, coinciding with the growing international policy focus on inclusive education (Salamanca Statement, see UNESCO, 1994).

More information on search terms, strategy and databases is presented in Supporting Information S1: Appendix S1. The search strategy was developed iteratively, drawing on terminology from key studies in the field (e.g., Gough Kenyon et al., 2020; Mandy et al., 2016; Rice et al., 2021) and refined through consultation with the research team to ensure comprehensive coverage across neurodivergent groups and transition‐related outcomes.

### Screening

Screening was undertaken independently by two researchers (V. S. and D. B.). All records identified through database searches were initially exported to Microsoft Excel spreadsheets. Duplicate records were removed using Excel's duplicate identification function followed by manual de‐duplication by both reviewers. Prior to the formal screening process, the reviewers piloted the screening procedure on a sample of five papers to ensure consistent interpretation of the inclusion and exclusion criteria. High agreement was achieved, after which the reviewers proceeded with full screening.

Titles and abstracts screening was conducted independently by V. S. and D. B. using the predefined eligibility criteria. A total of 488 articles were identified, 398 underwent title and abstract screening, with 51 progressing to full‐text review (see Figure 1). Full‐text screening was then conducted independently by the same two reviewers. Any disagreements during either stage of screening were initially resolved through discussion; where disagreements persisted, a third independent researcher (O. P.) was consulted to reach a final decision.

**FIGURE 1:**
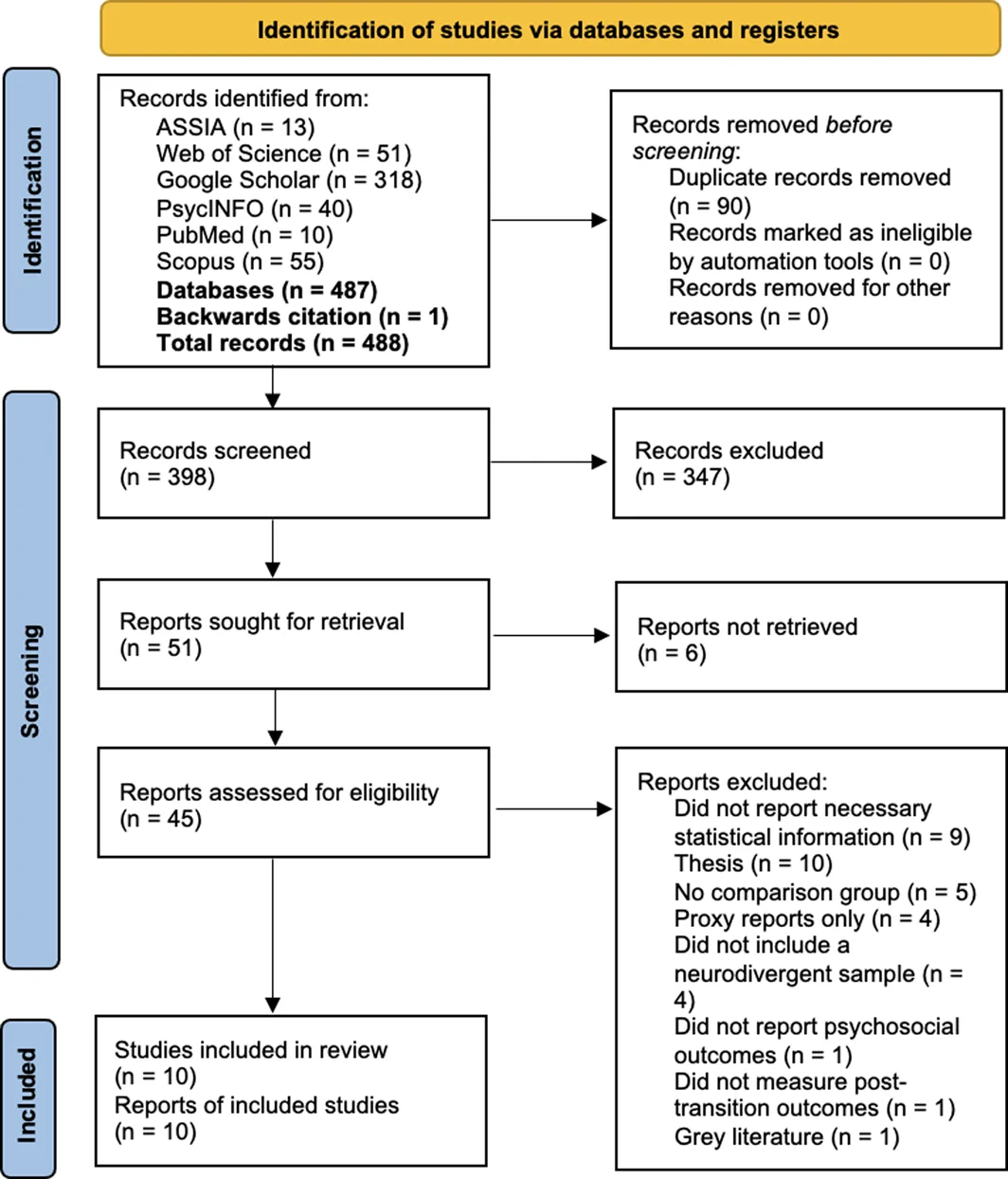
PRISMA flow diagram. PRISMA, preferred reporting items for systematic reviews and meta‐analyses.

A backward citation search was also conducted by V. S. to identify any additional relevant studies. One study (Hebron, 2017) was identified through this process but was subsequently excluded because it is a book chapter, and the same dataset was reported in a later peer‐reviewed publication (Hebron, 2018), which was retained. Similarly, Gough Kenyon et al. (2020) was excluded at full‐text screen because it reported pre‐transition data from the same cohort as Gough Kenyon et al. (2022), which was retained.

Inter‐rater reliability was assessed using Cohen's kappa. Agreement was high for both stages of screening, with *κ* = 0.93 for title and abstract screening and *κ* = 0.94 for full‐text screening, indicating excellent agreement according to the interpretation guidelines proposed by McHugh (2012).

Data extraction was conducted using a predefined extraction spreadsheet. Extracted data were subsequently double‐checked by a third researcher (O. P.) to ensure accuracy and consistency. Any discrepancies were resolved through discussion among the research team. In total, 10 studies met the eligibility criteria and were included in the review (see Table 1 for study characteristics and Table 2 for participant information).

**TABLE 1:** Study information.

Paper	Methodology	Outcome(s)	Measure(s)	Reporter	Effect	How established	Quality assessment/risk of bias
Dockrell and Lindsay (2007)	Quantitative & qualitative	Psychosocial outcomes (self‐esteem, social needs)	Standardised	Self‐reported	Negative	Significance testing	Low risk
Donaldson et al. (2025)	Quantitative	Emotional difficulties, conduct problems, hyperactivity, peer problems	Standardised	Self‐reported	Negative (mixed for gender)	Significance testing	Low risk
Hebron (2018)	Quantitative	School connectedness	Standardised	Self‐reported	Mixed (initial disadvantage for autistic, but gap reduced over time)	Significance testing	Low risk
Jelen et al. (2023)	Quantitative	Anxiety, depression	Standardised	Child and parent	Mixed due to disagreement between reporters (parent report showed negative effect of language; child report showed no significant effect)	Significance testing	Low risk
Gough Kenyon et al. (2022)	Quantitative	School concern, predictors of school concern	Standardised	Self‐reported	Negative (DLD significantly higher school concern; LL non‐significant	Significance testing	Low risk
Matthews et al. (2015)	Quantitative	Depression, anxiety, conduct problems	Standardised	Child and parent	Mixed (family and mental health factors predict isolation; early isolation does not predict later mental health once baseline symptoms are controlled)	Significance testing	Some concerns
Neal et al. (2016)	Quantitative	School and generalised anxiety	Study‐specific, non‐standardised	Child, parent, and teacher report	Mixed (systemic strategies reduced anxiety for typically developing children, not for SEN; other strategies non‐significant)	Significance testing	Low risk
Rice et al. (2021)	Quantitative	Pre‐transition: Transition concerns, expectations of settling into secondary school, academic attainment, mental health	Standardised	Child, parent, and teacher report	Mixed (teacher expectations strongest predictor; child concerns and parent expectations predictive for some outcomes)	Significance testing	Low risk
		Post‐transition: Academic attainment, classmate behaviour rating, school attachment, school liking, loneliness					
Vaz et al. (2014)	Quantitative	Academic competence, mental health functioning	Standardised	Self‐reported	Mixed (personal background factors, especially disability and SES, predicted AC and MHF; school contextual factors had limited predictive value; some changes across transition linked to school structure)	Significance testing	Low risk
Whelan et al. (2023)	Quantitative	Quality of life, student mental health, school belonging	Standardised	Child and parent	Mixed (autistic traits predicted QOL, mental health, and school belonging; child, family, and school factors contributed variably across outcomes)	Significance testing	Low risk

Abbreviations: AC, academic competence; DLD, developmental language disorder; LL, low language; MHF, mental health functioning; QOL, quality of life; SEN, special educational needs.

**TABLE 2:** Participant information in included studies.

Author	Country	Type of neurodivergence	Sample	SES	Age (years)	Gender	Ethnicity
Dockrell and Lindsay (2007)	England	DLD, SEN and neurotypicals	DLD (*n* = 69)	Not reported	8 and 10	DLD female: 17, DLD male: 52	Not reported
			SEN (*n* = 32)				
			Neurotypicals (*n* = 42)				
Donaldson et al. (2025)	England	Special educational needs	*n* = 1861	Eligibility for free school meals: Class 1 = 28.06%, class 2 = 19.54%, class 3 = 15.32%, class 4 = 12.13%	11.2 (baseline)	Male = 53.2%, female = 46.8%	White = 59.8%, Asian = 23.2%, Black = 7.5%, Mixed = 7.1%, Other = 2.4%
Hebron (2018)	England	Autism	Autistic (*n* = 28), neurotypical (*n* = 21)	Not reported	10–13	Autistic: Male = 23, female = 5; neurotypical: Male = 15, female = 5	Not reported
Jelen et al. (2023)	England	Language disorder	Year 1 *n* = 529, year 6 *n* = 384, year 8 *n* = 246	IDACI = 3908–32,471, mean rank = 21,351	Year 1 = 5–6, year 3 = 7–8, year 6 = 10–11, year 8 = 12–13	Year 6 LD: Female = 36 (45.0%); year 8 LD: Female = 21 (42.0%); year 6 no LD: Female = 141 (52.2%); year 8 no LD: Female = 95 (51.9%)	Not reported
Gough Kenyon et al. (2022)	England	DLD, low language	DLD (*n* = 30), LL (*n* = 29), neurotypical (*n* = 48)	Not reported	10–11	DLD: Male = 12, female = 18; LL: Male = 11, female = 18; neurotypical: Male = 26, female = 22	Not reported
Matthews et al. (2015)	England	ADHD, emotional or behavioural problems	*n* = 2232	Low = 33.2%	5–12	Not reported	Not reported
Neal et al. (2016)	England	Special educational needs	Neurotypical (*n* = 532), SEN (*n* = 89)	12.6% eligible for free school meals	11.21 (T1)	Male = 311, female = 310	37% minority ethnic
Rice et al. (2021)	England	Special educational needs	Wave 1: *n* = 744 children, *n* = 745 parents, *n* = 504 teachers; wave 2: *n* = 1712 children; wave 3: *n* = 1646 children	14.5% eligible for free school meals	10–11 (W1), 11–12 (W2), 11–12 (W3)	50% male	37% minority ethnic
Vaz et al. (2014)	Australia	Learning disability, attention deficit disorder, ADHD, autism	*n* = 266	Low, middle, and high income	11.89 (T1)	Male = 124, female = 142	Not reported
Whelan et al. (2023)	Australia	Autism, ADHD	*n* = 51	SEIFA, *M* = 1019, SD = 57	12.15	Male = 29 (56.9%)	Not reported

Abbreviations: ADHD, attention deficit hyperactivity disorder; DLD, developmental language disorder; IDACI, Income Deprivation Affecting Children Index; LD, language disorder; LL, low language; SEIFA, Socio‐Economic Indexes for Areas; SEN, special educational needs; SES, socio‐economic status; T, timepoint.

### Quality assessment

All papers that met the review inclusion criteria were assessed for quality using the quality appraisal checklist (IHE, 2014). In keeping with its correct usage, the checklist was contextualised for this review before being applied to the studies. Studies were rated across 16 domains, with responses of yes, partial/unclear, or no. Based on the proportion of items rated yes, overall risk of bias was categorised as low, some concerns, or high. Studies rated as low risk of bias were considered methodologically robust, though this does not necessarily indicate overall high quality, which also depends on factors such as sample size, generalisability, and outcome relevance. V. S. and D. B. scored the papers, see Table 1 for visualisation. Overall, no paper was rated as low quality/high risk. Matthews et al. (2015) was the only study that was flagged with some concerns around the design of the study, selection criteria as well as the lack of transparent reporting of variability and conflicts of interests. The remaining nine studies were rated as low risk.

### Data synthesis

Given the substantial heterogeneity in study design, outcome operationalisation, timing of assessment and regions, a meta‐analysis was not appropriate as it would have risked producing misleading summary estimates. In line with the recommended reporting standards for evidence synthesis without quantitative pooling, a narrative synthesis was therefore undertaken, with explicit attention to how methodological design influenced reported outcomes, following Synthesis without meta‐analysis guidelines (Campbell et al., 2020), as referenced in Supporting Information S1: Appendix S2 (PRISMA checklist). In reporting these results, it is important to acknowledge that the evidence base is overwhelmingly shaped by traditional neuro‐disorder oriented research paradigms rather than neurodiversity‐affirming or participatory approaches. None of the included studies were co‐produced with neurodivergent children or young people, nor did they report participatory involvement in the selection of outcomes, measures, or interpretation of findings.

## RESULTS

A total of 10 studies met the eligibility criteria and were included in the review. Full details of study characteristics, including methodology, outcomes, measures, reporters, and quality appraisal ratings, are presented in Table 1. Participant information, including country, neurodivergent group, sample size, age, gender, ethnicity, and socioeconomic indicators, is presented in Table 2. The PRISMA flow diagram illustrating the screening and selection process is presented in Figure 1.

All 10 studies included in the review employed quantitative designs, comprising nine prospective longitudinal studies that collected data spanning the primary to secondary school transition (Dockrell & Lindsay, 2007; Donaldson et al., 2025; Gough Kenyon et al., 2022; Hebron, 2018; Jelen et al., 2023; Rice et al., 2021; Vaz et al., 2014; Whelan et al., 2023), one longitudinal study measuring outcomes primarily post‐transition (Neal et al., 2016), and one population‐based birth cohort study examining associations between early language, social isolation and mental health across childhood and early adolescence (Matthews et al., 2015). Eight studies were conducted in England and two in Australia. Neurodivergent groups included autistic children, either with diagnosis or traits, children with DLD or specific LD, ADHD, and children identified under the broad umbrella of special educational needs.

Sample sizes range from *n* = 49 to *n* > 2000, with marked variation in analytic power. Outcomes were assessed using standardised measures, study specific‐instruments and all studies included child‐reported data on psychosocial and transition related concerns—some studies included also parent‐reported views.

### Impact of secondary school transition on child‐reported mental health outcomes

Overall, evidence regarding the impact of transition on general mental health outcomes in neurodivergent students was mixed and inconclusive. Across the 10 studies examining anxiety, depression, emotional difficulties and broader mental health functioning (MHF), no consistent pattern of deterioration attributable to transition was observed.

Four large‐scale cohort studies reported attenuated or null effects once baseline mental health and demographic factors were controlled, suggesting continuity of pre‐existing vulnerability rather than transition‐specific change (Donaldson et al., 2025; Jelen et al., 2023; Matthews et al., 2015; Rice et al., 2021). For example, in Matthews et al. (2015) social isolation was associated with later mental health difficulties, but early isolation did not independently predict post‐transition mental health once baseline symptoms were accounted for. Donaldson et al. (2025) reported elevated emotional and peer difficulties among students with special educational needs and disabilities (SEND) during transition, although effects varied by gender. This divergence indicates that study design could substantially influence detected effects.

In contrast, six smaller longitudinal studies (Dockrell & Lindsay, 2007; Gough Kenyon et al., 2022; Hebron, 2018; Neal et al., 2016; Vaz et al., 2014; Whelan et al., 2023) designed explicitly to capture the transition period more often reported heightened difficulties during or immediately following transfer. For instance, Gough Kenyon et al. (2022) and Dockrell and Lindsay (2007) showed that children with DLD reported poorer psychosocial outcomes, including self‐esteem, greater social needs, and higher levels of school concerns compared to their neurotypical peers.

### Impact of secondary school transition on child‐reported psychosocial outcomes

In contrast to general mental health outcomes, transition‐specific concerns emerged as one of the most consistent findings across studies. Two studies reported elevated levels of concern related to move to secondary school among neurodivergent students (Gough Kenyon et al., 2022; Rice et al., 2021). Two studies captured concerns during transition, but did not examine them explicitly (Dockrell & Lindsay, 2007; Donaldson et al., 2025). One study showed that children with DLD demonstrated higher levels of school concern than peers (Gough Kenyonet al., 2022). Similarly, another study highlighted that children with broader SEND reported that pre‐transition worries and expectations predicted selected post‐transition psychosocial outcomes (Rice et al., 2021). Analogous findings from a different study showed that by the spring of the first year of secondary school (Year 7 in England), both parents and their children with specific speech and language difficulties and other SEND reported difficulties around practicalities and managing academic work (Dockrell & Lindsay, 2007).

These findings were observed across different sample sizes and designs, suggesting that transition concern represents a proximal and methodologically robust indicator of psychosocial vulnerability, even when broader mental health outcomes appear mixed.

Evidence relating to school connectedness, belonging and adjustment was mixed but revealed consistent temporal pattern. Three studies (Hebron, 2018; Neal et al., 2016; Vaz et al., 2014) assessing outcomes shortly after transition reported initial disadvantages for autistic students and students with SEND. Hebron (2018) found that autistic students reported lower school connectedness during the early stages of secondary school compared to neurotypicals, though this gap reduced over time. This effect was replicated by Whelan et al. (2023) for school belonging and quality of life. Two studies (Rice et al., 2021; Whelan et al., 2023) with longer follow‐up periods observed partial convergence with neurotypical peers over time. This suggests that temporal sensitivity may be present and that cross‐sectional or delayed assessment may underestimate short‐term psychosocial disruption, while longer‐term designs may capture adaptation rather than initial transition impact.

In terms of peer relationships, social functioning, loneliness and social isolation were inconsistent. Some studies reported elevated peer difficulties during transition period, others demonstrated these associations were largely explained by baseline mental health or family factors. Both Donaldson et al. (2025) and Matthews et al. (2015) found that neurodivergent students reported peer and social functioning problems, but Matthews et al. (2015) noted that its predictive value diminished once baseline symptoms were controlled. After adjustment for pre‐transition symptoms, early peer difficulties did not consistently predict later mental health outcomes. This suggests that peer difficulties during transition from primary to secondary school may function as markers of ongoing vulnerability rather than independent transition effects.

### Patterns within and between specific groups of neurodivergent students

Gender was reported in most studies, nonetheless only one study examined gender as a moderator of transition‐related outcomes. Donaldson et al. (2025) reported gender differences in emotional and behavioural difficulties among students with SEND, with mixed patterns across outcomes. In terms of SES and other demographic factors, socio‐economic disadvantage was consistently associated with poorer psychosocial outcomes. For example, Vaz et al. (2014) found that disability status and SES were key predictors of MHF and academic competence, whereas school contextual factors showed limited predictive value. Yet, the moderating effect of SES was not examined in detail. Finally, reporting of ethnicity was inconsistent across studies and where reported, samples were predominantly white. No study examined ethnicity as a moderator of transition‐related mental health or psychosocial outcomes. Taken together, the evidence only supports synthesis of transition experiences by neurodivergent diagnostic group, while highlighting substantial gaps in examination of contextual influences. Despite these gaps, the available studies suggest distinct patterns of psychosocial adjustment across neurodivergent groups, with some studies indicating greater challenges related to anxiety, social adjustment, and school belonging during the transition to secondary school for certain neurodivergent groups compared to others. The small number of studies available within each neurodivergent group limited the ability to meaningfully synthesise findings or draw conclusions on specific neurodivergences.

### Impact of methodological design on observed outcomes

Consistently the evidence showed that the methodological design shaped the apparent impact of transition. Firstly, transition‐focused longitudinal studies and those assessing proximal experiences were more likely to detect psychosocial difficulties—highlighting that the follow‐up time‐point was a crucial factor in understanding mental health and psychosocial outcomes. Second, for studies in which both children and parents reported views, the parent‐reported outcomes tended to indicate greater difficulty than child self‐report, particularly for internalising symptoms. For studies in which children, parents and teachers reported, there were great inconsistencies again, but teacher reports seemed to be more justified on academic outcomes than social dimensions (Dockrell & Lindsay, 2007; Rice et al., 2021). This mirrors patterns observed in other areas of child mental health and highlights reporter choice as a key methodological determinant. Third, the large‐scale cohort studies, while they are methodologically robust, often diluted transition‐specific effects due to heterogeneity and emphasis on distal outcomes. Finally, the baseline adjustment frequently attenuated observed associations, suggesting that transition may amplify rather than cause vulnerability (Jelen et al., 2023; Rice et al., 2021).

## DISCUSSION

This review synthesised longitudinal evidence on mental health and psychosocial experiences of neurodivergent students during the transition from primary to secondary school. We aimed to identify both universal patterns across neurodivergent groups and group‐specific differences, emphasising outcomes grounded in children's self‐reports and in some cases lived experience rather than deficit‐based frameworks. A total of 10 studies met inclusion criteria, all incorporating neurotypical comparison groups, enabling contextualised interpretations of transition experiences. The literature is limited, not saturated, studies have heterogeneous samples and there is no gold standard guide for longitudinal studies to capture the effects of this transition.

The evidence on the mental health outcomes suggests that the transition to secondary school does not consistently precipitate new mental health difficulties. Instead, for some students, the transition may amplify pre‐existing vulnerabilities or make them more visible (Sideropoulos et al., 2024; Stack et al., 2020; Hebron, 2018). Evidence from children with dyslexia similarly highlights that mental health outcomes, including school‐related anxiety, are shaped more by the adequacy of learning support and classroom accommodations than by the transition itself (Wilmot et al., 2023, 2024). A pattern which aligns with the broader body of research across neurodivergent populations indicating that adolescent mental health outcomes are shaped by the interaction between individual characteristics and school environments. For example, Cheng et al. (2025) in a systematic review identified that negative school experiences lead to substantially higher emotional burden in children and young people with vision impairment. Pavlopoulou et al. (2025) also reported the same for autistic adolescents or those with ADHD. These findings highlight that contextual factors such as peer relationships, teacher interactions, and school climate play a key role in shaping mental health outcomes rather than these difficulties arising solely from within‐child characteristics. Finally, while these outcomes may differ in intensity from neurotypical peers, it is important to emphasise that the transition is a period of adjustment for many students; neurodivergent children often face challenges due to environmental misalignment, lack of support, or limited anticipation of their needs (Fielding et al., 2025; Fisher et al., 2025).

Psychosocial outcomes appear to be more sensitive indicators of transition‐related challenges than diagnostic mental health outcomes, capturing experiences that are immediate, contextual and developmentally salient. Measures such as school concern, belonging, connectedness, peer difficulties, and self‐esteem consistently detected transition‐related challenges, particularly in the early post‐transition period. Our findings are consistent with those of Donaldson et al. (2025) and Dockrell and Lindsay (2007), who reported that children with DLD and SEND found the transition more difficult, experienced lower levels of self‐esteem and encountered challenges with the curriculum and organisation of the school—challenges often not affecting neurotypical peers. However, Gough Kenyon et al. (2022) found that school concerns decreased between the end of primary school and the beginning of secondary school, then plateaued for the rest of the year for children with DLD, suggesting that the early post‐transition period may be the critical window for intervention.

Finally, our review highlights notable heterogeneity in experiences across neurodivergent populations. While some patterns are shared, such as early post‐transition peer and belonging difficulties, the magnitude and nature of these experiences vary depending on the specific neurodivergence, co‐occurring conditions, and individual and environmental factors. Comparison, at times, across neurodivergent groups illuminates both shared vulnerabilities and divergence in experiences, emphasising the need for tailored approaches that consider individual profiles, contextual supports, and intersecting factors such as age, gender, ethnicity, and socio‐economic background (Cheng et al., 2025; Villatoro et al., 2018). Notably, what remains absent from this evidence base is as informative as what is present—children with Williams Syndrome and DS were not represented in any study meeting our inclusion criteria, and where data does exist for these groups, it relies on parent report rather than child self‐report (Sideropoulos et al., 2024).

### Whose knowledge is represented

From a neurodiversity‐informed perspective, the findings of this review must be interpreted within the context of whose knowledge is represented, and whose is absent. Although several studies included child self‐report data, the research questions, outcome frameworks and analytic interpretations remained researcher‐led and grounded in traditional developmental and psychopathology models. This constrains the extent to which conclusions can be said to reflect neurodivergent priorities, values or definitions of wellbeing. The scarcity of research coproduced by and for different groups of neurodivergent students is concerning—we do not know from young people themselves what they consider a priority, or what their preferred methods of inquiry may be. Addressing this is essential not only as a methodological consideration but as an ethical one: you cannot design effective support systems without hearing from the children themselves.

### Limitations and challenges

Several methodological limitations constrain the current evidence base. Most of the included studies were conducted in the UK, limiting generalisability to other international contexts. However, comparable patterns have been reported in Australia, USA, and other countries (Anderson et al., 2016; Carpendale et al., 2025; Lundqvist et al., 2019). Sample sizes were frequently small, and reliance on traditional questionnaires may not fully capture the experiences of neurodivergent students (e.g., measures like Strengths and Difficulties Questionnaire are known for ceiling effects in neurodivergent populations, Iizuka et al., 2010). Few studies were co‐produced with children or incorporated participatory methods, meaning that available data largely reflects researcher‐led conceptualisations of mental health and psychosocial adjustment. Collecting data directly from neurodivergent students, particularly those that have been excluded from research such as children with Williams Syndrome, using mixed methods and participatory approaches is crucial for understanding priorities and experiences that matter most to young people themselves (Palikara et al., 2018). Furthermore, many of the studies in the area are cross‐sectional and retrospective (Hughes et al., 2013; Maras & Aveling, 2006), making it difficult to reliably examine changes over time.

Although most schools in the UK offer some formal transition programmes, there is great variation in the aims and the content (Evangelou et al., 2008), and studies examining the effectiveness are rare. Many focus on resilience and development of coping mechanisms but are not co‐produced and co‐delivered with neurodivergent children and young people (Mandy et al., 2016) while others are grounded in neuro‐disorder narratives and may not include neurodivergent students' perspective on how to live their best life in school (Kakoulidou et al., 2023). Hughes et al. (2013) highlighted the importance of future transition research employing individual differences models, as even children with the same type of neurodivergence, may require different levels of support given the variability in their profiles of needs and strengths (Maras & Aveling, 2006). Finally, some included reports may draw on overlapping samples: Neal et al. (2016), Rice et al. (2021) and Donaldson et al. (2025); all report on pupils from South Easy England followed across the transition. As each examined different outcomes, all were retained, but our findings should be interpreted with this possible overlap in mind.

### Implications for intervention: Towards a neurodiversity model of support

Effective support for neurodivergent students during transition to secondary school should emphasise environmental fit, relational continuity, and flexible accommodations rather than remediation of individual deficits. Interventions should be co‐produced with neurodivergent students and responsive to the heterogeneity within and across neurodivergent groups. Evidence from children with dyslexia suggests that anxiety and depression are linked to low connectedness to school as a whole rather than to dyslexia itself (Wilmot et al., 2023, 2024). It is well established that neurodivergent children require differentiated teaching approaches, modified to consider their specific needs. Such adaptations are commonplace in therapeutic anxiety interventions for vulnerable populations, such as for autistic students (Lang et al., 2010; Moree & Davis, 2010) and are also likely to be appropriate when intervening to reduce anxiety across secondary transition. This personalised approach seems particularly important given evidence that neurodivergent children often face barriers—including the expectation to adapt their communication and social approaches to meet neurotypical norms, which can erode self‐esteem over time—that are likely to compound vulnerability during transition (e.g., heightened risk of bullying, social exclusion) (Dykens, 2000; Evangelou et al., 2008; McGreevy et al., 2026; Terras et al., 2009). It also aligns with a broader neuro‐affirmative, transdiagnostic, and participatory approach to child and adolescent mental health research (Bellato & Seker, 2025), and extends the ‘stage–environment fit’ theory (Midgley et al., 2014), which proposes that positive developmental outcomes are most likely when educational contexts align with young people's evolving needs. Applied through a neurodiversity lens, this framework foregrounds the responsibility of schools to adapt their practices, expectations and structures, rather than requiring neurodivergent students to adapt to inflexible systems. This is particularly significant given that school transitions represent precisely the kind of bounded, anticipated life event during which proactive, well‐timed support can meaningfully shape longer‐term developmental and mental health trajectories (Donaldson et al., 2023).

### Future directions

Future research must adopt a person‐centred approach, employing a combination of standardised measures and co‐produced tools and interviews appropriate for neurodivergent people. A collaborative approach to whole‐school wellbeing that involves young people, school staff and parents/carers offers opportunities for researchers to deliver knowledge exchange initiatives and supports that attune more genuinely to the needs and priorities of students (Kakoulidou et al., 2023). Longitudinal coproduced designs hold particular value for research in this area—by tracking neurodivergent children over an extended time, they offer insights into educational, developmental, and mental health trajectories, as well as the factors that shape outcomes. Comparing different neurodivergences within such designs allows for a better understanding of the unique and shared impact of school transitions and accommodates informed transition planning (Sonuga‐Barke et al., 2024). Addressing this knowledge gap is imperative to better understand the overrepresentation of neurodivergent students in mental health, social care and youth justice systems, and advocate effectively for their needs during this crucial educational shift.

## CONCLUSION

The findings of this review highlight that the transition to secondary school is not inherently harmful for neurodivergent students, but that poorly structured, inflexible, and unsupportive school environments can make it so. Longitudinal, coproduced research that addresses different forms of neurodivergence is essential for identifying the individual and contextual factors that determine wellbeing and mental health during this transition. The application of a neurodiversity lens, foregrounding environmental responsibility, lived experience, and the active involvement of the entire school community remains underexplored, and represents a promising direction for research and evidence‐based practices in schools.

## AUTHOR CONTRIBUTIONS

**Olympia Palikara**: Conceptualization; investigation; writing—original draft; methodology; validation; supervision; writing—review and editing; project administration. **Rebecca Lucas**: Conceptualization; investigation; methodology; validation; supervision; writing—review and editing. **Danielle Baptiste**: Data curation; formal analysis; writing—review and editing. **Cat Jones**: Writing—review and editing; data curation; formal analysis. **Laura Kelly**: Writing—review and editing; formal analysis; data curation. **Georgia Pavlopoulou**: Writing—original draft; writing—review and editing; formal analysis; data curation. **Vassilis Sideropoulos**: Conceptualization; investigation; writing—original draft; writing—review and editing; project administration; data curation; supervision; resources; formal analysis; validation; visualization; methodology.

## CONFLICT OF INTEREST STATEMENT

The authors declare no conflicts of interest.

## ETHICAL CONSIDERATIONS

This study is a systematic review of previously published literature and did not involve the collection of primary data from human participants. As such, informed consent or assent was not required, and ethical approval from a Research Ethics Committee or Institutional Review Board was not applicable.

## AI STATEMENT

The Authors declare that they have not used AI in any capacity that would require disclosure in accordance with Wiley's AI Guidelines, nor in any capacity that would reasonably require discloser for editors, reviewers and readers to properly evaluate their research or manuscript. The authors confirm that AI has not been used for purposes including but NOT limited to drafting and editing, to generate substantial text or restructure arguments, nor in the research methodology. The authors take full responsibility for the accuracy of this statement.

## REGISTRATION DETAILS

This systematic review was conducted in accordance with PRISMA 2020 guidelines and was pre‐registered on the Open Science Framework (OSF) (Registration number: 9R384; Date of registration: 11 June 2025). The registration is available at https://doi.org/10.17605/OSF.IO/9R384.

## Supporting information

Supporting Information S1

## Data Availability

Data sharing not applicable to this article as no datasets were generated or analysed during the current study.

## ENDNOTE

^1^
For the purposes of this review, the outcomes were interpreted broadly to capture the wide range of constructs examined in the transition literature. These included indicators related to emotional wellbeing, psychological distress (e.g., anxiety, depressive symptoms), social adjustment (e.g., peer‐relationships, belonging, school connectedness), and perceived adjustment to the new school environment.
